# COVID‐19 and the increase in schizophrenia incidence in the future: A hypothesis and a serious warning

**DOI:** 10.1002/hsr2.978

**Published:** 2022-12-05

**Authors:** Mohammad Pourfridoni, Hedyeh Askarpour

**Affiliations:** ^1^ Student Research Committee Jiroft University of Medical Sciences Jiroft Iran; ^2^ Clinical Research Development Center of Imam Khomeini Hospital Jiroft University of Medical Sciences Jiroft Iran

**Keywords:** COVID‐19, incidence, schizophrenia

## Abstract

**Background and Aims:**

The coronavirus disease 2019 (COVID‐19), which has caused a global pandemic, is brought on by the Acute Respiratory Syndrome coronavirus 2 (SARS‐CoV‐2). Since the COVID‐19 pandemic started so recently, dealing with complications that emerge years later and have the potential to cause several crises for humanity is one of the issues we face in the post‐COVID‐19 age. Therefore, we wish to discuss a theory and potential dangers surrounding the probability of schizophrenia following COVID‐19 infection in this study.

**Methods:**

The literature search for this article has been entirely internet‐based. Information was gathered using the Web of Science, PubMed, Scopus, and Google Scholar databases.

**Results:**

The results showed that multiple immune system changes brought on by COVID‐19 have been identified as potential causes of schizophrenia.

**Conclusion:**

It is predicted that one of the long‐term effects of COVID‐19 is an increase in the risk of schizophrenia incidence based on the results of this study, which looked at the pathophysiology and etiology of schizophrenia as well as the pathogenic mechanisms of the SARS‐CoV‐2. Therefore, healthcare staff should be prepared to handle any potential risks in future.

## 1 BACKGROUND

The severe acute respiratory syndrome coronavirus 2 (SARS‐CoV‐2) is the culprit behind the coronavirus disease of 2019 (COVID‐19), leading to a pandemic worldwide. Globally, this pandemic has caused serious problems, and our knowledge of this disease is still developing.^1^ Our understanding of the short‐ and long‐term complications of SARS‐CoV‐2 infection is continuously expanding as our experience with this virus evolves. Various studies have reported neurological and neuropsychiatric complications in patients with COVID‐19.^2^, ^3^ Considering that not much time has passed since the start of the COVID‐19 pandemic and also that this disease is mysterious, one of the challenges we face in the post‐COVID‐19 era is to meet the complications that manifest themselves years later and it may cause many crises for humanity. Therefore, in this study, we want to propose a hypothesis and possible threats about the possibility of schizophrenia incidence following COVID‐19 infection so that health activists around the world can prevent possible risks by working on this issue and being ready to face the possible dangers.

## 2 SCHIZOPHRENIA

Delusional beliefs, hallucinations, and changes in thought, perception, and behavior are all characteristics of the functional psychotic disorder schizophrenia. Positive symptoms, such as hallucinations, delusions, and formal thought disorders, and negative symptoms, such as anhedonia, poverty of speech, and lack of desire, have traditionally been separated into two primary categories.^4^ Schizophrenia is a severe, commonly appearing mental disorder that poses a challenge for both etiology and treatment. It is marked by psychotic symptoms and frequently leads to social and occupational impairment.^5^ Schizophrenia can cause serious problems that affect every aspect of life. Suicide, suicide attempts, thoughts of suicide, anxiety disorders, obsessive‐compulsive disorder (OCD), depression, inability to work or attend school, social isolation, health, and medical problems, and victimization are some of the complications that schizophrenia may lead to or be related with.^6^ Despite having a low prevalence, schizophrenia has a huge global disease burden.^7^

### 2.1 Pathophysiology

Regarding schizophrenia developments, there are three primary theories. According to the neurochemical abnormality hypothesis, an imbalance of dopamine, serotonin, glutamate, and GABA causes the disease's psychiatric symptoms. The presence of anomalies in the cerebral structure, the absence of gliosis, which suggests in utero changes, and the finding that motor and cognitive impairments in patients occur before the commencement of the illness are additional indications that schizophrenia is a neurodevelopmental disorder. The disconnect hypothesis, on the other hand, emphasizes the changes in neuroanatomy. Schizophrenia results in a decrease in grey matter volume that affects both the parietal and temporal lobes.^7^

### 2.2 Etiology

The etiology of schizophrenia has not been clearly defined. According to several studies, alterations in numerous neurotransmitters, such as glutaminergic and GABA hypoactivity or dopaminergic, serotonergic, and alpha‐adrenergic hyperactivity, contribute to the development of schizophrenia. Genetics also has a substantial impact.^7^ One of the causes of schizophrenia may be inflammatory changes and an imbalance of cytokines.^8^ Research on the etiology of schizophrenia has long focused on infection and immunity. Numerous studies have discovered that prenatal exposure to bacterial, viral, and *Toxoplasma gondii* infections increases the risk of schizophrenia.^9^, ^10^ Viral infection has been identified as one of the environmental risk factors for schizophrenia in epidemiological research.^11^ In those who are genetically predisposed, prenatal and perinatal infections may have an impact on the immune response or brain developmentt and lead to schizophrenia. There is a T helper (Th) 1/Th2 imbalance in untreated schizophrenia patients, with suppressed levels of Th1‐related cytokines and compensatory raised Th2‐cytokine levels. The primary cell type in preserving immunological homeostasis, including the balance of the innate and adaptive immune systems, is the regulatory T cell (Treg) cell. Tregs primarily control the neuroimmune interaction between astrocytes and microglia in the central nervous system (CNS). In schizophrenia, impaired Treg cells cause astroglial overactivation and microglial pruning. In the development of Treg cells, interleukin (IL)‐2 is crucial.^12^

## 3 COVID‐19 AND SCHIZOPHRENIA

Initially, it was assumed that COVID‐19 only had an impact on respiration. However, rising evidence reveals that COVID‐19 is also associated to a variety of neurological disorders. Patients with COVID‐19 may experience neurological symptoms as a result of a cytokine storm and an intensified state of inflammation.^13^

Although the pathogenic processes causing CNS invasion are yet unknown, it appears that SARS‐CoV‐2 enters the brain via a hematogenous pathway. Alternative routes, such as the cribriform plate of the ethmoid bone close to the olfactory bulb, should be taken into account. In addition, the isolation of SARS‐CoV‐2 from cerebrospinal fluid implies that the virus might directly infect the neurological system and harm nerves.^14^

The severe, occasionally deadly pulmonary and cardiac consequences of COVID‐19 infection are believed to be caused by a profound inflammatory response, and it has been hypothesized that this response also triggers neuropsychiatric symptoms via immunological processes. In the pathophysiology of psychiatric diseases including schizophrenia, depression, and psychotic disorders, viruses' infection, and immune‐based triggers have long been thought to play a role. C‐reactive protein has been investigated as a potential peripheral measure of immunological activation, which is suggested to have a causative or triggering function in schizophreniform psychosis. Human coronaviruses have been demonstrated to possess neuroinvasive properties as a result of either autoimmune or viral replication, leading to the hypothesis that they may function as opportunistic infections of the CNS. In fact, additional coronaviruses have been associated with neuroinflammation and CNS penetration in psychotic disorders.^15^ Also, COVID‐19 can cause many inflammatory changes in the body; including disruption of the Th1/Th2 balance, changes in the amount of Tregs, and disruption of the IL2 balance.^16^

Eventually, it can be said that COVID‐19 can cause several changes in the human immune system, which are known as possible etiology of schizophrenia. Besides the biological effects of COVID‐19, which can increase the risk of schizophrenia, the COVID‐19 pandemic may be contributing to an increase in reactive psychotic disorders since it is so stressful.^17^ This may be explained by diathesis‐stress hypothesis. In this theory, stressful experiences have always been thought of as one of the key contributors in the development and exacerbation of mental disorders, regardless of the impact of nervous system infection. One of the longest pathoetiological hypotheses for schizophrenia was the interaction between outside stimuli and internal susceptibility. Additionally, according to this theory, psychosocial stress may induce the microglia to become pathologically activated, which could result in excessive synaptic pruning and the loss of cortical gray matter. Therefore, damage to the stress‐sensitive area may cause negative cognitive symptoms to arise immediately. Additionally, a loss of cortical control may prevent subcortical dopamine from acting, resulting in the positive symptoms of psychosis.^18^ Therefore, since not much time has passed since the beginning of the COVID‐19 pandemic, it is possible that in the not‐too‐distant future we will see cases of schizophrenia that were previously infected with the SARS‐CoV‐2. This possibility is more for people who have encountered this virus during the prenatal and perinatal periods. We have had a similar experience of this happening in such a way that after influenza epidemics, people got schizophrenia due to exposure to this virus. But because COVID‐19 has a much wider pandemic and is also unknown and shows new aspects of itself day by day, it scares us more.

## 4 CONCLUSION

Overall, we should think about conducting studies that investigate the incidence of schizophrenia due to COVID‐19, and also health activists should be prepared for possible threats. Previous studies have shown that COVID‐19 has various psychosocial effects on schizophrenia patients, which makes these patients show worse mental recovery and worsen their symptoms such as stress, anxiety, fear, sadness, and loneliness. Thus, schizophrenic patients should be specially cared for and supported against COVID‐19. As a result, it is suggested to conduct more studies on the psychosocial symptoms of COVID‐19 on schizophrenia patients at the same time as studying the incidence of schizophrenia following COVID‐19.^19^

## AUTHOR CONTRIBUTIONS

**Mohammad Pourfridoni**: Conceptualization; writing – original draft; writing – review and editing. **Hedyeh Askarpour**: Writing – review and editing.

## CONFLICT OF INTEREST

The authors declare no conflict of interest.

## TRANSPARENCY STATEMENT

The lead author Hedyeh Askarpour affirms that this manuscript is an honest, accurate, and transparent account of the study being reported; that no important aspects of the study have been omitted; and that any discrepancies from the study as planned (and, if relevant, registered) have been explained.

## Data Availability

Data sharing not applicable to this article as no datasets were generated or analysed during the current study. Data sharing is not applicable to this article as no new data were created or analyzed in this study.
